# A Novel *BIRC6* Variant Impairs Apoptotic Regulation in Familial Premature Ovarian Insufficiency: Functional Validation in a CRISPR/Cas9 Zebrafish Model

**DOI:** 10.1007/s43032-026-02088-6

**Published:** 2026-05-04

**Authors:** Mahsa Kazerani, Umut Cagiral, Seyedeh Zoha Tabatabaei, Remziye Nur Akalper, Ugur Bora, Sadegh Babashah, Gunes Ozhan, Mehdi Totonchi

**Affiliations:** 1 Department of Molecular Genetics, Faculty of Biological Sciences, Tarbiat Modares University, P.O. Box: 14115-154, Tehran, Iran; 2 Izmir Biomedicine and Genome Center (IBG), Dokuz Eylul University Health Campus, Balcova, 35340 Izmir Türkiye; 3 Izmir International Biomedicine and Genome Institute (IBG-Izmir), Dokuz Eylul University, Balcova, 35340 Izmir Türkiye; 4 Cardiogenetic Research Center, Rajaie Cardiovascular Medical and Research Institute, Iran University of Medical Sciences, Tehran, Iran; 5 Department of Molecular Biology and Genetics, Izmir Institute of Technology, Urla, 35430 Izmir Türkiye; 6 Department of Genetics, Reproductive Biomedicine Research Center, Royan Institute for Reproductive Biomedicine, ACECR, Tehran, Iran

**Keywords:** Premature ovarian insufficiency, Whole exome sequencing, Baculoviral IAP Repeat-Containing Protein 6, CRISPR/Cas9, Zebrafish model

## Abstract

Premature ovarian insufficiency (POI), characterized by ovarian dysfunction before age 40, remains idiopathic in over 50% of cases, underscoring the urgent need to elucidate its genetic underpinnings. Here, we report a consanguineous Iranian family with five females diagnosed with POI, exhibiting elevated gonadotropins, undetectable anti-Müllerian hormone, and bilateral ovarian atrophy. Whole Exome Sequencing identified a novel homozygous missense variant in *BIRC6* (NM_016252.4: c.11266C > T; p.Arg3756Cys), a gene encoding an apoptosis regulator. Segregation analysis confirmed an autosomal recessive inheritance pattern, with homozygosity exclusively in affected individuals. Functional studies in a CRISPR/Cas9-generated *birc6*^*del12*^ zebrafish model recapitulated POI phenotypes: homozygous females exhibited reduced fecundity, aberrant oocyte morphology, and elevated embryonic death. Transcriptional analysis revealed dysregulation of apoptotic (*badb*, *sortilin*, *apc*) and fertility-related (*nanos1*, *vtg1*) genes, alongside unaltered estradiol levels, implicating apoptosis-driven follicular atresia rather than endocrine dysfunction. Compensatory increase in egg production in mutants mirrored human POI progression, where initial irregular cycles culminate in follicular depletion. This study establishes *BIRC6* as a novel POI candidate, links its anti-apoptotic function to ovarian homeostasis, and highlights zebrafish as a tractable model for dissecting POI mechanisms. Our findings expand the genetic landscape of infertility and suggest therapeutic potential for apoptosis modulation in fertility preservation.

**Supplementary Information:**

The online version contains supplementary material available at 10.1007/s43032-026-02088-6.

## Introduction

Infertility, as defined by the World Health Organization (WHO), is the inability to achieve spontaneous conception after ≥ 12 months of unprotected sexual intercourse, affecting both male and female reproductive systems. Recent WHO estimates reveal that approximately 17.5% of the worldwide adult population suffers from infertility, with prevalence rates differing across regions and ethnic groups [1]. Premature ovarian insufficiency (POI), historically termed premature ovarian failure (POF), is a critical contributor to female infertility, characterized by the cessation of ovarian function before age 40. POI affects approximately 3.7% of women under 40 and is diagnosed clinically by oligomenorrhea/amenorrhea, elevated follicle-stimulating hormone (FSH) levels (> 25 IU/L), and hypoestrogenism, confirmed via two serum tests ≥ 4 weeks apart [2]. Despite advances in diagnostic criteria, the etiology of POI remains idiopathic in over 50% of cases, suggesting that there must be further research into its complex pathogenesis [3]. POI has a substantial genetic foundation, with chromosomal anomalies and identified POI-associated genes constituting roughly one-third of instances. However, in most patients, the underlying genetic cause remains unknown [4, 5]. To date, Whole Exome Sequencing (WES) has identified more than 20 pathogenic variants in POI-associated genes, including *FIGLA* (folliculogenesis), *STAG3* (meiotic cohesion), and *FSHR* (hormone signaling), which are implicated in critical pathways such as oocyte maturation, DNA repair, and apoptotic regulation [6–8] (Table 1). Familial aggregation studies reveal a sixfold increased POI risk among first-degree relatives, supporting a strong hereditary component [9, 10]. Elucidating the molecular mechanisms governing ovarian folliculogenesis—such as follicular recruitment, maturation, and atresia—may enable the development of gene-targeted therapies to rescue or delay ovarian dysfunction [11]. Ongoing efforts to integrate WES with functional genomics (e.g., transcriptomic profiling) promise further unraveling of the pathogenesis of POI and expanding precision medicine applications [12].

**Table 1:** Summary of selected candidate genes implicated in primary ovarian insufficiency

Gene	Biological Role	Location	POI Mechanism	Variant Type	Ref
AMHR2	AMH type II receptor; regulates follicular recruitment	12q13.13	Loss-of-function → altered follicle recruitment kinetics	Missense	[13]
ATG7	Autophagy protein 7; autophagy induction	3p25.3	Heterozygous mutations → impaired autophagy → germ cell loss	Missense, frameshift	[14]
BMP15	Oocyte-derived TGFβ ligand; regulates granulosa cell proliferation	Xp11.2	Reduced BMP15 activity → impaired oocyte-granulosa signaling → follicle growth arrest	Missense, frameshift, nonsense	[15, 16]
ESR2	Estrogen receptor β; mediates estrogen signaling in granulosa cells	14q23.3	Missense mutations → altered estrogen sensitivity → impaired follicle maturation	Missense	[17]
FANCA	Fanconi anemia complementation protein; mediates HR and ICL repair	16q24.3	Biallelic mutations → impaired interstrand crosslink repair → meiotic defects	Frameshift, nonsense, missense	[18, 19]
FIGLA	bHLH transcription factor; oogenesis-specific; regulates zona pellucida genes	2p13.3	Loss-of-function → decreased zona pellucida gene expression → follicle dysgenesis	Frameshift, missense, nonsense	[20, 21]
FOXL2	Pleiotropic transcription factor; maintains GC identity throughout development	3q23	Missense mutations → GC reprogramming/differentiation defect → follicle dysgenesis	Missense, frameshift	[22, 23]
FSHR	FSH receptor; regulates follicular development and estrogen synthesis	2p16.3	Biallelic loss-of-function → impaired FSH signaling → ovarian resistance to FSH	Missense, frameshift, nonsense	[16, 24]
GDF9	Oocyte-derived TGFβ ligand; cumulin with BMP15; promotes GC proliferation	5q11.2	Biallelic mutations → impaired cumulin signaling → reduced FSH sensitivity	Missense, frameshift, nonsense	[16, 25]
HFM1	DNA helicase; mediates homologous recombination and synapsis	1p22.1	Biallelic missense mutations → impaired HR → meiotic defects → oocyte loss	Splice site, missense	[26]
MCM8	Helicase; MCM8-MCM9 complex mediates HR and DNA synthesis	20q13.2	Biallelic loss-of-function → impaired HR → genome instability → oocyte apoptosis	Frameshift, missense, nonsense	[27]
MCM9	Helicase; MCM8-MCM9 complex mediates HR and DNA synthesis	6p21.32	Biallelic loss-of-function → impaired HR → meiotic defects → POI	Frameshift, nonsense, missense	[27]
MSH4	MutS homolog; MSH4-MSH5 dimer stabilizes chromosome pairing	1p31.1	Biallelic mutations → impaired synapsis & meiotic recombination → oocyte loss	Frameshift, splice site, missense	[28, 29]
NANOS3	RNA-binding protein; represses apoptosis in germ cells	19q13.4	Variants → reduced germ cell survival → reduced follicle pool	Missense, frameshift	[30]
NOBOX	Oocyte homeobox transcription factor; early folliculogenesis regulator	7q35	Loss-of-function → impaired oocyte gene expression → reduced primordial follicles	Frameshift, nonsense, missense	[16, 31]
NOTCH2	Notch receptor; regulates GC proliferation	1p12	Missense mutations → altered Notch signaling → impaired GC proliferation	Missense	[32]
REC8	Meiosis-specific cohesin subunit; maintains sister chromatid cohesion	3p23	Mutations → impaired cohesion → meiotic arrest → oocyte loss	Frameshift, missense	[33, 34]
SOHLH1	Basic HLH transcription factor; regulates oogenesis and primordial follicle development	9q34.3	Loss-of-function → impaired follicle development → reduced follicle pool	Missense, frameshift	[35]
STAG3	Cohesin subunit; maintains sister chromatid cohesion during meiosis	7q22.1	Loss-of-function → impaired synapsis & cohesion → meiotic arrest → oocyte loss	Frameshift, nonsense, missense	[8, 36]
TP63	p53 family transcription factor; controls oocyte DNA damage response	3q28	Missense mutations (C-terminal) → enhanced TAp63 activity → excessive apoptosis	Missense, frameshift, duplication	[37, 38]

This study reports a novel homozygous missense variant in the Baculoviral IAP Repeat-Containing Protein 6 (*BIRC6/BRUCE)* gene (NM_016252.4: exon55: c.11266C > T: p.Arg3756Cys; rs367790330), identified in a consanguineous Iranian family using whole-exome sequencing. The *BIRC6* gene, located on chromosome 2q37.3, encodes a multifunctional protein that belongs to the inhibitor of apoptosis protein (IAP) family. The produced protein suppresses programmed cell death by directly connecting with executioner caspases (e.g., caspase-3 and caspase-7) and facilitating their ubiquitin-mediated degradation, obstructing apoptotic signals [39, 40]. Beyond its canonical anti-apoptotic role, the BIRC6 protein contributes to autophagy regulation, cytokine signaling modulation (e.g., the NF-κB pathway), and the DNA damage response, where it coordinates repair mechanisms to maintain genomic stability [41–43]. These pleiotropic functions underscore BIRC6’s central role in cellular homeostasis and stress adaptation. Considering that tightly regulated apoptosis is critical for oocyte survival and follicular maintenance in the ovary, genes like *BIRC6* that safeguard cell viability represent biologically plausible candidates for POI pathogenesis [5, 42]. The discovered variant represents the first association of the *BIRC6* gene with POI, expanding the genetic landscape of ovarian dysfunction. Segregation analysis confirmed its recessive inheritance pattern, and in silico tools (e.g., MutationTaster, CADD) predicted strong pathogenicity, consistent with the hypergonadotropic hypogonadism phenotype reported in the afflicted family members.

Genetic analysis in the affected family strongly supports this variant as a plausible cause of the disease. To move beyond association and more firmly establish causality, we used a zebrafish model to functionally assess how this variant affects relevant biological pathways and contributes to the observed phenotype. Zebrafish (*Danio rerio*) have emerged as a premier model for biological research due to their genetic resemblance to humans (70%−80%), external fertilization, and short generation time of approximately 3 months. Importantly, zebrafish share conserved molecular and cellular pathways governing oogenesis, folliculogenesis, and apoptotic regulation with mammals, making them highly suitable for reproductive and ovarian biology studies. Their ability to produce hundreds of embryos per clutch, together with optical transparency during early development, permits real-time visualization and quantitative assessment of oocyte development, follicular dynamics, and early embryonic outcomes and folliculogenesis, making them a valuable model for fertility research [44–46]. Moreover, zebrafish are particularly well suited for CRISPR/Cas9-mediated genome editing, enabling efficient generation of targeted loss-of-function alleles and rapid functional interrogation of candidate disease genes implicated in human infertility [47, 48]. Zebrafish models have been widely used to study ovarian failure, follicle depletion, and apoptosis-driven reproductive phenotypes, providing a robust in vivo platform to dissect conserved mechanisms underlying POI [49, 50].

In this work, we developed the first *birc6* knockout zebrafish line to model the POI pathogenesis. This genetically tractable system enables high-throughput investigation of *BIRC6* function in oocyte survival, regulation of apoptosis, and folliculogenesis, processes that are evolutionarily conserved across vertebrates. Our phenotypic analysis uncovered ovarian hypoplasia, impaired reproductive output, and increased embryonic loss in mutant females, capturing key aspects of ovarian dysfunction relevant to human POI. Together, these findings establish zebrafish as a suitable and informative model for investigating *BIRC6*-associated ovarian pathology and provide a foundation for future studies aimed at fertility preservation and targeted therapeutic development.

## Patients, Materials, and Methods

### Patient Recruitment and Clinical Evaluation

**Fig. 1:**
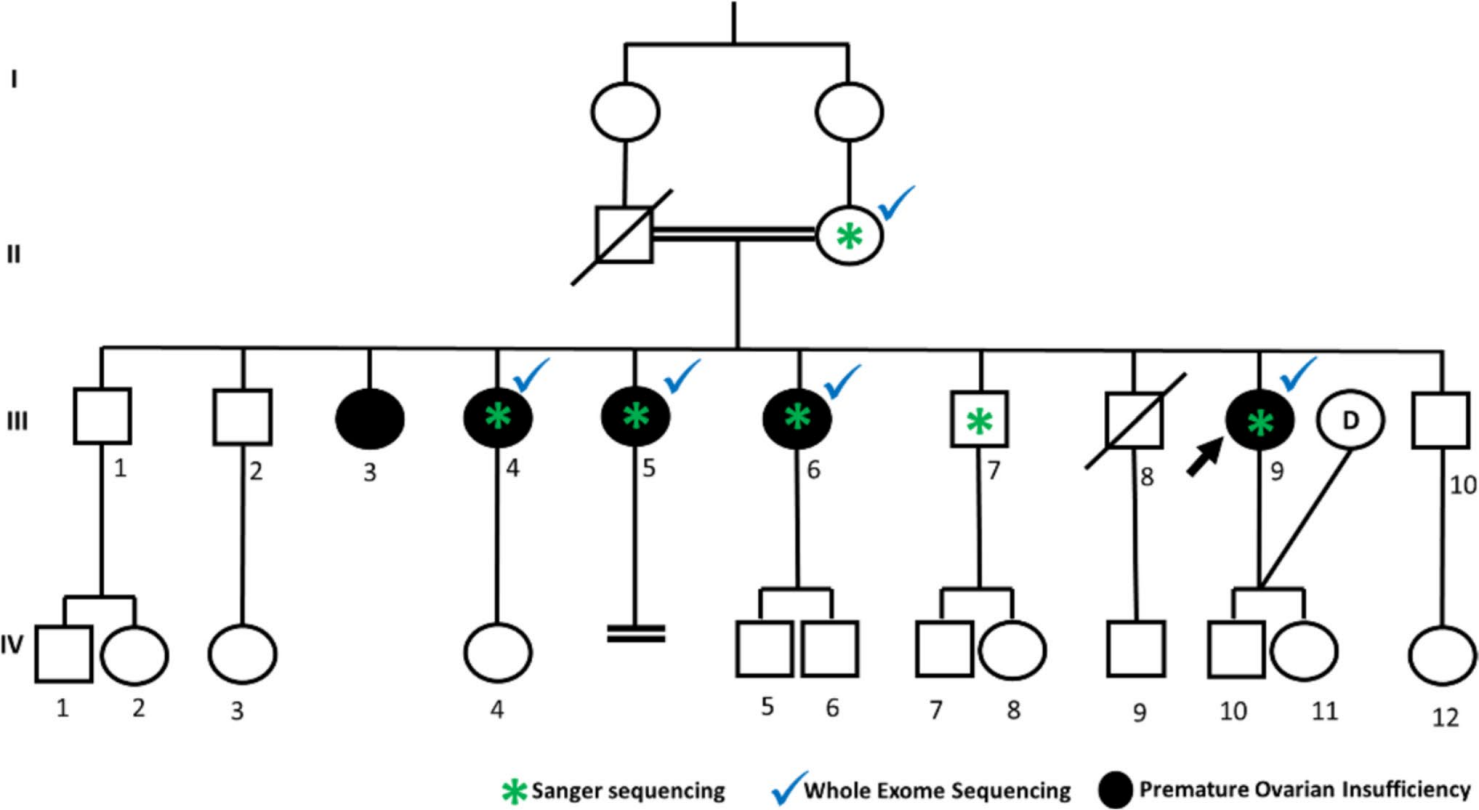
Pedigree of a consanguineous Iranian family with autosomal recessive premature ovarian insufficiency (POI). Affected individuals (black symbols) are homozygous for the *BIRC6* variant (NM_016252.4: c.11266C > T; p.Arg3756Cys). The proband (III-9, arrow) and her four sisters (III-3, III-4, III-5, and III-6) exhibit POI. Parents (II-1 and II-2) are first cousins, denoted by a double horizontal line. Unaffected siblings are shown as open symbols (circles: females; squares: males). All affected females presented with menopause before age 40, while males showed normal fertility. Individual III-3 was unmarried and disagreed to take part in the research project

Throughout this study, we recruited a consanguineous Iranian family with five affected females who were all diagnosed with POI. The proband (III-9), a 38-year-old woman, presented with four years of unexplained infertility following a first-cousin marriage. Consistent with genetic counseling, her four sisters (III-3, III-4, III-5, and III-6) similarly exhibited POI. There was no associated family history of premature menopause or reproductive abnormalities among the parents, who were first cousins. Diagnostic evaluations complied with the European Society of Human Reproduction and Embryology (ESHRE) guidelines. The diagnostic workup included karyotyping to exclude chromosomal abnormalities, ultrasonography to assess ovarian volume and antral follicle count as well as uterine morphology, *FMR1* premutation screening via CGG repeat analysis, and comprehensive hormonal profiling. Following inconclusive results from standard diagnostic testing, the family underwent WES to identify potential monogenic causes of their condition (Fig. 1). An overview of the experimental workflow, including human genetic analyses and zebrafish functional experiments, is provided in Supplementary Figure S1.

### DNA Sample Preparation and Whole Exome Sequencing

Peripheral blood samples were collected from four affected family members (III-4, III-5, III-6, and III-9), one male sibling (III-7), and their unaffected mother for molecular analysis (II-2). Genomic DNA was extracted from leukocytes using the standard salting-out procedure. The purity of the DNA was assessed through A260/A280 and A260/A230 ratios, and its quantity was measured with a NanoDrop spectrophotometer (Thermo Fisher Scientific, USA). Exome libraries were prepared using the Agilent SureSelect XT Enrichment System (Agilent Technologies, USA), with the SureSelect Human All Exon V5 probe set for exome capture. Sequencing was performed on the Illumina HiSeq 4000 platform (Illumina, USA) with paired-end 150 bp reads, achieving an average coverage depth of 100 × across the exome. Raw sequencing data (FASTQ files) underwent quality control using FastQC to assess read quality and adapter contamination [51]. Adapter trimming and quality filtering were performed with Trimmomatic [52]. The cleaned reads were aligned to the human reference genome (GRCh38/hg38) using the Burrows-Wheeler Aligner (BWA-MEM, v0.7.17) [53]. Duplicate reads were marked, and base quality score recalibration (BQSR) was performed using the Genome Analysis Toolkit (GATK, v4.2.0.0) [54]. Variant calling was conducted with GATK’s HaplotypeCaller (v4.2.0.0) in GVCF mode, followed by joint genotyping across all samples [55]. The identified variants were then annotated for functional impact, population frequency, and pathogenicity predictions using ANNOVAR and the Variant Effect Predictor from Ensembl. An annotated VCF file was generated for all sequenced samples. Given the consanguineous pedigree and autosomal recessive POI pattern, homozygous variants were prioritized and filtered by MAF < 0.01 (gnomAD v4.1.0, ExAC, 1000 Genomes, Iranome), protein-altering impact (missense, nonsense, frameshift, canonical splice), deleterious predictions (CADD ≥ 20, DANN ≥ 0.9), and damaging predictions from in silico tools such as SIFT4G, MutationTaster, LRT, and ClinPred. Finally, we restricted analysis to ovarian-expressed genes or those previously implicated in ovarian biology or female infertility, consistent with WES-based POI studies.

Final variant interpretation was performed using the Franklin (Genoox) web platform (https://franklin.genoox.com), which integrates population, computational, and functional evidence in accordance with the 2015 ACMG/AMP guidelines. For the variant described in this report, Franklin indicated fulfillment of ACMG/AMP criteria PM2 and PP2 (pathogenic evidence) together with BS2 (benign supporting), leading to an overall classification as a variant of uncertain significance (VUS).

### Segregation Analysis by Sanger Sequencing

Sanger sequencing was performed on all accessible family members (II-2, III-4, III-5, III-6, III-7, and III-9) to confirm the candidate *BIRC6* variation (NM_016252.4: exon55: c.11266C > T: p.Arg3756Cys; rs367790330). The Oligo 7.0 program (Molecular Biology Insights, Colorado Springs, CO, USA) was utilized to design primer pairs that include exon 55 of *BIRC6* (Forward: 5′-GCCGACCTAGTTGCTCCTATT-3′; Reverse: 5′-AGGCACTCAACCCTCACTAA-3′). Polymerase chain reaction (PCR) was performed using a thermal cycler (Applied Biosystems, USA) under the following conditions: an initial denaturation at 95 °C for 5 min, followed by 31 cycles of amplification consisting of denaturation at 95 °C for 25 s, annealing at 62 °C for 25 s, and extension at 72 °C for 35 s. A final extension was carried out at 72 °C for 7 min. Following variant amplification, the purified PCR products were bidirectionally sequenced using the BigDye Terminator v3.1 on an ABI 3500 system (Applied Biosystems, Foster City, CA, USA). The resulting electropherograms were analyzed and matched with the reference sequence (NM_016252.0) using FinchTV v1.4.0 (Geospiza, Seattle, WA, USA).

### Zebrafish Handling and Maintenance

Every experiment involving zebrafish (Danio rerio) was carried out in compliance with ARRIVE guidelines for animal research and protocols authorized by the Institute of Biomedical Genetics' Animal Experiments Local Ethics Committee (IBG-AELEC). Embryos were reared in E3 embryo medium (containing 5 mm NaCl, 0.33 mm MgSO₄, 0.33 mm CaCl₂, 0.17 mm KCl, and methylene blue; pH 7.2) [56]. The larvae were moved to a recirculating aquaculture system in the IBG Zebrafish Core Facility at 5 days post-fertilization (dpf), where they were kept at a density of ≤ 5 fish/L. The adult zebrafish were kept at 28 °C with a 12-h light/dark cycle and continuous biological and mechanical filtration. Fish were fed a standardized diet: three times daily until 3 months post-fertilization (mpf), followed by twice-daily feedings of granular commercial feed. Sexually mature females were fasted for 24 h before embryos were collected for the experiment. When necessary, human euthanasia was performed using 168 mg/L tricaine methanesulfonate (MS-222; Sigma-Aldrich, USA) buffered with 336 mg/L sodium bicarbonate, followed by rapid cooling on ice, in compliance with AVMA guidelines. One-cell-stage embryos were microinjected with 1 nL of ribonucleoprotein complexes (Cas9 protein, sgRNA, and tracer dye) using borosilicate glass needles (Sutter Instrument, USA) and a pneumatic PicoPump injector (World Precision Instruments, USA) to perform CRISPR/Cas9-mediated mutagenesis. To prevent pigmentation, embryos were incubated in E3 medium supplemented with 0.003% 1-phenyl-2-thiourea (PTU) after injection [57].

### RNA Extraction and Quantitative Real-Time PCR

Total RNA was isolated from zebrafish embryos or dissected gonads using QIAzol lysis reagent (QIAGEN, Germany) and purified with the RNeasy Micro Kit (QIAGEN, Germany) according to the manufacturer’s instructions. To preserve RNA integrity, tissues were homogenized in QIAzol immediately after dissection [58]. RNA purity and concentration were assessed using a NanoDrop spectrophotometer (Thermo Fisher Scientific, Wilmington, DE, USA). Only samples with A260/A280 ratios between 1.8 and 2.1 and A260/A230 ratios > 2.0 were used for downstream analysis. For all samples, equal RNA input (1 µg) was used for first-strand cDNA synthesis using the ProtoScript II First Strand cDNA Synthesis Kit (New England BioLabs, MA, USA) with oligo(dT) primers and ProtoScript II Reverse Transcriptase [59]. The resulting cDNA was diluted 1:10 in nuclease-free water prior to quantitative real-time PCR (qRT-PCR). qRT-PCR was performed using SYBR Green Master Mix (Applied Biosystems, CA, USA) on an ABI Step One Detection System (Applied Biosystems, USA). Each reaction was run as one biological replicate with three technical replicates each. Relative gene expression levels were calculated using the 2^−ΔΔCt^ method, normalized to the zebrafish ribosomal protein L13a (*rpl13a*) gene, which has been validated as a stable reference gene in zebrafish embryos and adult tissues [58, 60–62]. Gene selection was guided by the established role of BIRC6 (Baculoviral IAP Repeat Containing 6) in the mitochondrial apoptosis pathway (KEGG pathway: map04215) and previous studies linking apoptotic regulation to ovarian function. Accordingly, expression of mitochondrial pro-apoptotic genes was assessed in embryos, while fertility- and oogenesis-related markers were analyzed in gonadal tissue.

### CRISPR/Cas9-Mediated Genome Editing in Zebrafish

A zebrafish knockout line for *birc6* was generated using CRISPR/Cas9-mediated genome editing to introduce a 12-bp deletion in exon 55 of the gene. The guide RNA (gRNA) target sequence was selected using the CRISPRscan algorithm (https://www.crisprscan.org/gene/) to maximize on-target efficiency and minimize potential off-target effects. The selected gRNA sequence (5′-GGCCACTGTGGCATTCTCGA-3′) was chosen based on its high predicted activity and the absence of closely related genomic sites, with the most similar potential off-targets containing at least three to four mismatches. Considering this design and prior experience with CRISPR workflows in our laboratory, no additional off-target screening validation was performed [59, 63].

The gRNA template was generated by PCR using a forward primer containing the T7 RNA polymerase promoter sequence at the 5′ end followed by the 20-nt target-specific region and a universal reverse primer encoding the ~ 80-bp constant gRNA scaffold, together with a universal Cas9 scaffold template and Q5 High-Fidelity DNA Polymerase (New England Biolabs, MA, US). The resulting PCR product was used for in vitro transcription using the HiScribe T7 Quick High Yield RNA Synthesis Kit (New England BioLabs, MA, US). The transcribed gRNA was purified with the NucleoSpin Gel and PCR Clean-up Kit (Macherey–Nagel, Germany), followed by RNA Clean and Concentrator-25 (Zymo Research, CA, US) as described previously [63]. The injection mixture contained 600 ng/µL gRNA and 300 ng/µL Guide-it Recombinant Cas9 protein (Takara Bio Inc., Japan), supplemented with 0.05% phenol red. Approximately 1–1.5 nL of this solution was injected into one-cell stage embryos.

For generation of the F0 founders, Cas9/gRNA was injected into ~ 200 embryos, from which 24 larvae were randomly selected and screened for mutagenesis. Positively edited individuals were raised and crossed to wild-type fish to obtain F1 heterozygotes. A similar screening strategy was applied to the F1 generation, and two confirmed heterozygous carriers were selected to generate the F2 generation. In the F2 generation, 24–30 fish were screened, and homozygous *birc6*^*del12*^ mutants were identified and separated from heterozygous and wild-type siblings. A total of 10 homozygous *birc6*^*del12*^ fish were established, of which 6 were females and used for downstream phenotypic and molecular analyses. High-resolution melting (HRM) analysis was used for initial mutation screening, and the precise 12-bp deletion was confirmed by Sanger sequencing, as described in the corresponding subsections below.

### Genomic DNA Extraction and Sanger Sequencing

Genomic DNA was isolated from either whole larvae (5 days post-fertilization, dpf) or tail fin clips of adult zebrafish [63]. Tissue samples were lysed in 100 µL of extraction buffer (10 mM Tris–HCl pH 8.0, 50 mM KCl, 0.3% Tween 20, 1 mM EDTA) by incubation at 98 °C for 10 min. Proteinase K (20 µg/mL; Thermo Fisher Scientific, Waltham, MA, USA) was added, followed by overnight incubation at 55 °C and subsequent heat inactivation at 98 °C for 10 min. Lysates were centrifuged at 12,000 × *g* for 5 min, and supernatants were transferred to fresh tubes. DNA was precipitated by adding 50 µL nuclease-free water, 10 µL 3 M sodium acetate (pH 5.2), and 180 µL ice-cold isopropanol. After incubation on ice for 10 min, samples were centrifuged at 12,000 × *g* for 15 min at 4 °C. Pellets were washed with 500 µL of 70% ethanol, air-dried, and resuspended in 30 of µL nuclease-free water. DNA concentration and purity were assessed using a NanoDrop spectrophotometer (Thermo Fisher Scientific, MA, USA).

### High-Resolution Melting Analysis and Sanger Sequencing

High-resolution melting (HRM) analysis was used as the primary screening method to identify CRISPR/Cas9-induced mutations in the birc6 locus, followed by Sanger sequencing for definitive validation. Primers were designed using the NCBI Primer-Blast tool (https://www.ncbi.nlm.nih.gov/tools/primer-blast/) to amplify a 115 base-pair (bp) region encompassing the CRISPR/Cas9 target site in the *birc6* locus. The primer sequences were as follows: Forward: 5′-GTCATCAAGCCGCAGTAGTGG-3′ and Reverse: 5′-CATGAGCCTCTGGTTGTTTGG-3′. For the initial F0 generation, genomic DNA was extracted from 24 embryos/fish selected from approximately 200 Cas9/gRNA-injected embryos and subjected to HRM screening. HRM-positive samples were subsequently validated by Sanger sequencing to confirm the presence and nature of induced mutations. The same strategy was applied to the F1 generation, in which HRM-positive heterozygous fish were again confirmed by Sanger sequencing before being selected for breeding. For the F2 generation, 24–30 fish per clutch were screened by HRM to identify homozygous mutants. Because HRM profiles did not reliably distinguish homozygous wild-type from homozygous mutant alleles, all HRM-positive samples were further analyzed by Sanger sequencing. Ultimately, 10 homozygous *birc6*^*del12/del12*^ fish were identified, of which six were females, and these individuals were used for downstream experiments.

For HRM analysis, genomic DNA was diluted to 2 ng/µL and loaded into each well of a 96-well LightCycler plate (Sarstedt Ag & Co., Germany). HRM was performed using a LightCycler 480 Instrument II (Roche Diagnostics, Switzerland). Samples were initially heated to 95 °C for 2 min, followed by PCR amplification cycles of 95 °C for 10 s and 60 °C for 30 s. After amplification, melting analysis was carried out by heating to 95 °C at a ramp rate of 0.02 °C/s, followed by cooling to 40 °C. Melting curves were analyzed using LightCycler 480 software v1.5.1.62 to distinguish wild-type, heterozygous, and mutant profiles.

For Sanger sequencing, PCR products spanning the birc6 target region were purified using the ExoSAP-IT PCR Cleanup Reagent (Thermo Fisher Scientific) and sequenced bidirectionally on an ABI 3500xL Genetic Analyzer (Applied Biosystems, CA, USA) with BigDye Terminator v3.1 chemistry. Electropherograms were analyzed using SnapGene v6.0 (Insightful Science, CA, USA) and aligned to the reference *birc6* sequence (GRCz11/danRer11). The 12-bp deletion (c.11266_11277del) was confirmed in homozygous *birc6*^*del12/del12*^ mutants, while wild-type and heterozygous samples exhibited intact sequences or overlapping peaks, respectively.

### Mating Assay

After establishing the homozygous *birc6*^*del12/del12*^ line, mating assays were performed using female mutants (*n* = 6) and compared with four wild-type (wt-AB) breeding pairs. For each mating, one *birc6*^*de112/del12*^ female was paired with one wt-AB male, while wt controls consisted of one WT female paired with one WT male. Male *birc6*^*de112*^ mutants were also tested in preliminary matings but showed no significant phenotype and were therefore not included in further fertility analyses. Breeding was conducted weekly over a 10-week period using the same labeled fish pairs to minimize inter-individual variability. Fish were maintained in standard breeding tanks containing only the fish and a barrier placed over an elevated strainer for embryo collection. Feeding and decorative plants were excluded from breeding tanks to avoid effects on spawning behavior. Tanks were kept visually isolated from each other to prevent partner confusion. A transparent divider separated males and females overnight; the next morning, the divider was removed to allow 1 h of spawning. All embryos were collected, and the total number of eggs and necrotic embryos were recorded on Day 0 and Day 1 after spawning. For each clutch, all embryos (typically 80–300 per mating, depending on fecundity) were analyzed as a single biological replicate. To assess developmental status, embryos were examined under a stereomicroscope and compared with stage-matched WT embryos according to the standard zebrafish developmental staging criteria [64]. Embryos were classified as (i) normal, when the developmental stage and morphology matched WT embryos at the same time point; (ii) delayed, when embryos showed slower progression but retained overall normal morphology; and (iii) abnormal, when embryos exhibited clear morphological defects (e.g., irregular cleavage, abnormal body axis, malformed somites, edema, or developmental arrest). Embryo viability was further categorized as alive or necrotic at each time point. The numbers of normal, delayed, abnormal, and necrotic embryos were recorded for each clutch and used for subsequent analyses. To prevent unintended use of experimental fish, WT fish included in the study were labeled as “Prohibited” in the facility throughout the experimental period, and mutant fish were housed separately with individual labels.

### Enzyme-Linked Immunosorbent Assay

For estradiol measurements, the same homozygous *birc6*^*de112/del12*^ females (*n* = 6) and wt-AB females from the mating assay were analyzed. Each female was placed in a separate breeding tank with one wt-AB male and equal water volume. Tanks were kept in an open box allowing normal light–dark cycling while maintaining complete isolation of each pair and preventing visual contact between tanks. Estradiol levels in breeding water were measured using a competitive Estradiol ELISA Kit (#501890, Cayman Chemical, MI, USA). Although the kit is designed for serum/plasma samples, direct blood collection from zebrafish proved impractical due to low volume, poor sample quality after tail clipping, and loss of animals. Therefore, breeding water was used as a non-invasive alternative. Prior to the experimental measurements, a pilot validation was performed using wild-type fish under identical breeding conditions, which demonstrated that the colorimetric signals obtained from water samples fell within the dynamic range of the kit’s standard curve. Only after confirming assay feasibility was the analysis extended to the *birc6*^*del12/del12*^ group. All steps were carried out according to the manufacturer's instructions, with water used as the sample matrix. Absorbance was read at 414 nm using a Multiskan GO spectrophotometer (Thermo Scientific, MA, US). Measurements were performed using the same biological replicates as in the mating assay (*n* = 6 per group), and samples were analyzed in technical duplicates.

### Hematoxylin and Eosin Staining

For histological analysis, homozygous *birc6*^*del12/del12*^ females (*n* = 6) and age-matched wt-AB females (*n* = 4) were euthanized after completion of the mating assays. Fins and heads were removed, and the remaining bodies were fixed in 10% neutral buffered formalin for 3 days at room temperature [65]. Following fixation, samples were decalcified in 0.35 M EDTA for 4 days, with daily solution changes. Tissues were then dehydrated through a graded ethanol series (70%, 80%, 90%, 96%, and 100% EtOH), each for 1–2 min, with final dehydration in absolute ethanol. After dehydration, samples were cleared in xylene (three changes, 40–60 s each) and embedded in paraffin. Paraffin blocks were sectioned at 5 µm thickness using a microtome, and sections were mounted on glass slides. For deparaffinization, slides were incubated in xylene (2 × 15–20 min), followed by rehydration through a descending ethanol series (100%, 96%, 80%, and 70% EtOH) and rinsed in distilled water. Sections were stained with hematoxylin for 3–6 min, rinsed in running tap water, differentiated briefly in acid alcohol (70% EtOH with HCl), and blued in ammonia water. After washing, sections were counterstained with eosin for 1–5 min, followed by dehydration through ascending ethanol series and clearing in xylene (2 × 5–10 min). Finally, slides were mounted with coverslips using a permanent mounting medium and examined under a light microscope for ovarian morphology and follicular features.

### Statistical Analysis

All statistical analyses were performed using GraphPad Prism 8 (GraphPad Software, USA). Data are presented as mean ± SEM unless otherwise stated. Sample sizes (*n*) and statistical tests used are indicated in the figure legends. For mating assays, four *birc6*^*del12*^ females (confirmed by Sanger sequencing) were each paired with wild-type males, and four wild-type mating pairs served as controls. Egg production and embryo outcomes were recorded weekly over a 10-week period using the same mating pairs. Fecundity percentage and estradiol levels were analyzed using unpaired two-tailed Student’s t-tests**.** Clutch size (number of eggs laid) was analyzed using the Mann–Whitney U test due to non-normal data distribution and frequent zero values. Embryo survival and death percentages were analyzed using a mixed-effects model to account for repeated measurements over time. Egg counts in ovaries were analyzed using an unpaired two-tailed Student’s t-test after assuming parametric distribution. Gene expression analyses were performed using Student’s t-test on ΔCt values, with three technical replicates per sample. Due to limited availability of fertile mutant fish, biological replicates were not available for qPCR experiments, which represents a limitation of the study. A *p*-value < 0.05 was considered statistically significant. Data are presented as mean ± standard deviation (SD) or median with interquartile range (IQR), as appropriate.

## Results

### Clinical Description of the Proband

The proband, a 38-year-old woman, was referred to reproductive endocrinology for evaluation of primary infertility and a history of irregular menstrual cycles progressing to secondary amenorrhea. Comprehensive clinical and genetic assessments, conducted in accordance with the ESHRE diagnostic criteria for POI, revealed normal body mass index (BMI: 22.1 kg/m^2^), external genitalia, intellectual function, karyotype (46, XX), and FMR1 gene CGG repeat count (< 40 repeats). Transvaginal ultrasound demonstrated bilateral ovarian atrophy (right ovary: 1.2 cm^3^; left ovary: 1.1 cm^3^) with no antral follicles observed. Hormonal profiling indicated marked elevation of follicle-stimulating hormone (FSH: 36.4 IU/L) and luteinizing hormone (LH: 21.1 IU/L), alongside reduced anti-Müllerian hormone (AMH: 0.3 ng/mL) level and decreased estradiol amount (87.1 pg/mL), consistent with a diagnosis of POI. In the context of age-adjusted reference ranges for reproductive-age women (typically around 1–3 ng/mL), an AMH level of 0.3 ng/mL indicates markedly diminished ovarian reserve. (Table 1). The proband was born to consanguineous parents (first-cousin marriage). Familial evaluation identified four elder sisters (III-3, III-4, III-5, and III-6), all of whom experienced menopause before age 40, suggesting a familial predisposition to POI. Individual III-3 reported no history of marriage or pregnancy. Sisters III-4 and III-6 achieved uncomplicated natural pregnancies, while III-5 underwent two unsuccessful IVF cycles and remains infertile. The proband’s brothers (*n* = 5) exhibited normal fertility, with children conceived through natural pregnancies. During the study period, the proband conceived twice via donor oocyte in vitro fertilization (IVF). Her first pregnancy resulted in a tubal ectopic gestation requiring surgical intervention. Subsequently, she successfully delivered two healthy term infants (Fig. 1). No affected individuals in the pedigree reported histories of pelvic surgery, chemotherapy, radiation exposure, autoimmune disorders, or endocrinopathies (e.g., thyroid dysfunction, adrenal insufficiency). Systemic evaluations ruled out metabolic and infectious contributors to ovarian insufficiency.

### Identification of a Novel *BIRC6* Variant Using Whole Exome Sequencing

Whole exome sequencing (WES) was performed on five family members (II-2, III-4, III-5, III-6, and III-9), achieving an average coverage of 100 × and > 93% of reads with a quality score ≥ 30 (Q30), fulfilling primary quality control thresholds. Initial analysis generated an annotated variant call format (VCF) file for all samples. Given the consanguineous parentage and autosomal recessive inheritance suspected in all affected daughters, we prioritized variants present in the homozygous state within autozygous regions—a strategy shown to markedly reduce the search space and increase causal variant yield in consanguineous families. In this study, compound heterozygous variants were not systematically pursued beyond standard quality and frequency filtering, and structural variants (e.g., CNVs) were not comprehensively assessed, a known limitation of short-read WES. A homozygous missense variant in the *BIRC6* gene (NM_016252.4: exon55: c.11266C > T: p.Arg3756Cys; rs367790330) was identified in all four affected sisters. In silico pathogenicity predictions yielded conflicting results: SIFT4G, LRT, MutationTaster, PrimateAI, MetaRNN, M-CAP, CADD, FATHMM-MKL, BayesDel, AlphaMissense, and ClinPred classified the variant as deleterious. Conversely, FATHMM, FATHMM-XF, MVP, MetaLR, MetaSVM, REVEL, PROVEAN, and SIFT predicted tolerance. Evolutionary conservation analysis revealed high conservation of the altered residue (GERP + + _NR = 5.5; GERP + + _RS = 3.34). For this variant, the Franklin classification fulfilled ACMG/AMP criteria PM2 and PP2 (pathogenic evidence) together with BS2 (benign supporting), resulting in a variant of uncertain significance (VUS). Segregation data supported the ACMG/AMP framework as PP1_moderate, as all homozygous females manifested POI and no unaffected female relative was homozygous. The MAF in gnomAD v4.1.0 was 0.00002752 (2/72,644 alleles), with no occurrences in the Iranome database (800 Iranian controls). The absence of the variant in ethnically matched controls underscores its rarity and potential relevance to the observed familial premature ovarian insufficiency (POI). The identified *BIRC6* variant (rs367790330) has been submitted to ClinVar (accession: SUB15965848) for public archival and clinical interpretation.

### Sanger Sequencing Validation and Segregation Analysis

**Fig. 2:**
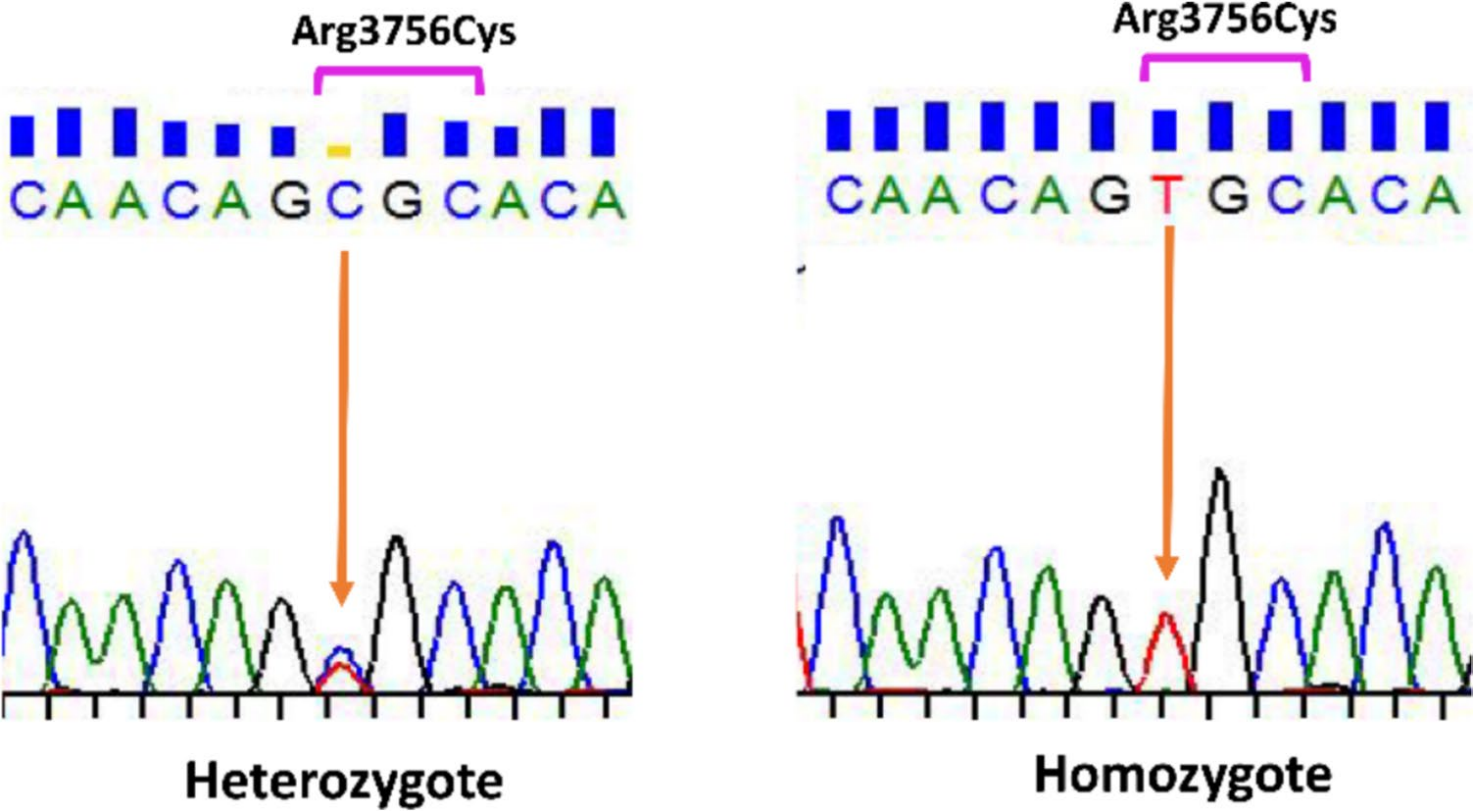
Sanger sequencing validation of the *BIRC6* variant in a consanguineous family with premature ovarian insufficiency (POI). Electropherograms confirm homozygosity for the *BIRC6* c.11266C > T (p.Arg3756Cys) variant in four affected females (III-4, III-5, III-6, III-9; arrows) and one unaffected male sibling (III-7). Their mother (II-2) is heterozygous (C/T), consistent with autosomal recessive inheritance. The variant (T/T genotype) is absent in control sequences (C/C, not shown). POI manifests exclusively in homozygous females, while hemizygous male (III-7) retains normal fertility, underscoring the sex-specific phenotypic expression of this variant

Sanger sequencing, the gold standard for orthogonal validation of WES findings, confirmed homozygosity for the *BIRC6* variant (NM_016252.4: exon55: c.11266C > T: p.Arg3756Cys; rs367790330) in all four affected sisters and one unaffected brother (Fig. 2). Their unaffected mother was heterozygous for the variant, consistent with an autosomal recessive inheritance pattern. The segregation analysis further demonstrated complete concordance between genotype and phenotype, with homozygosity observed exclusively in females exhibiting POI.

### Structural and Functional Implications of the *BIRC6* Variant

**Fig. 3:**
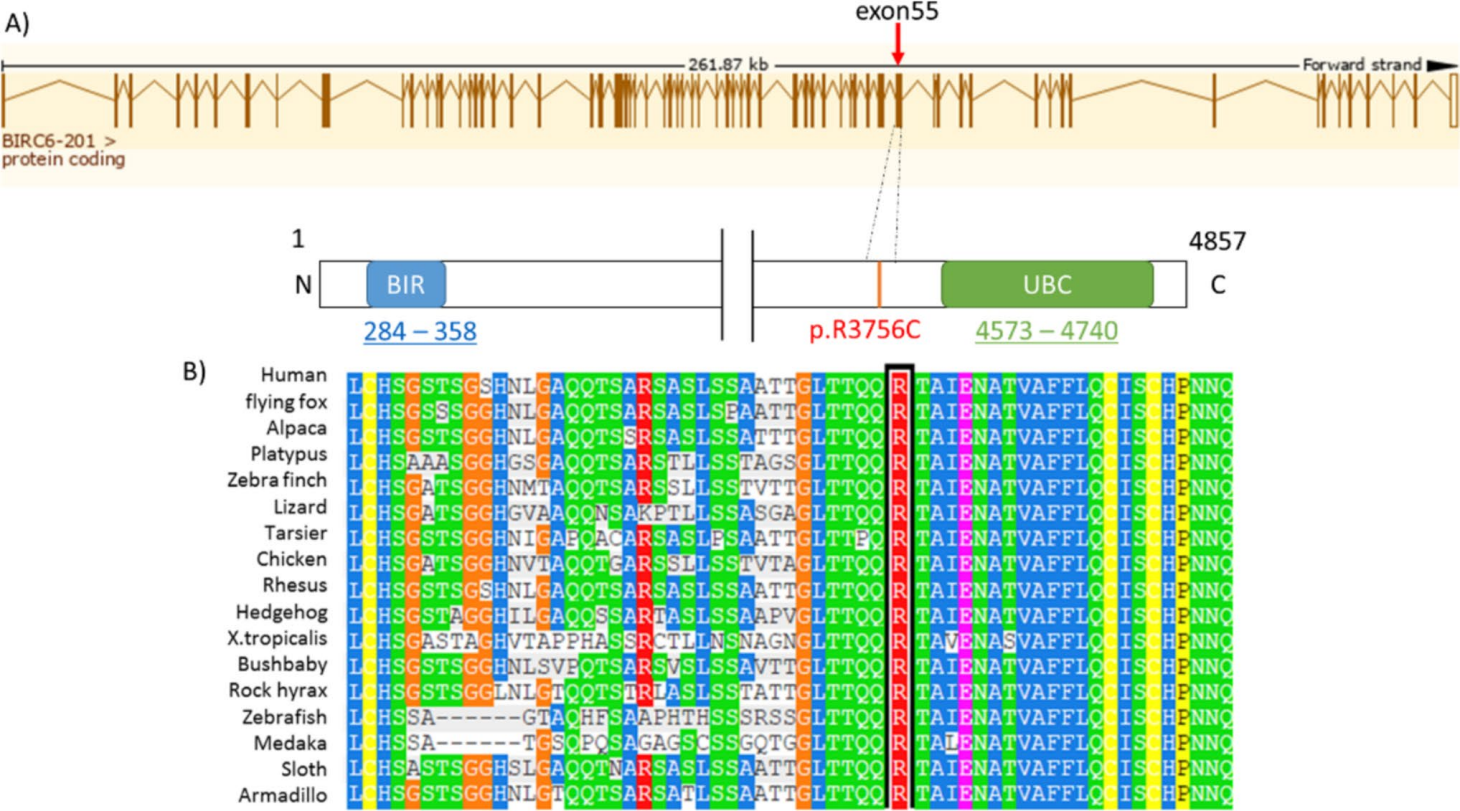
Structural and evolutionary analysis of the *BIRC6* p.Arg3756Cys variant. (**A**) Schematic of the human *BIRC6* transcript (NM_016252.4) depicting functional domains based on Ensemble: baculovirus IAP repeat (BIR, blue) and ubiquitin-conjugating (UBC, red). The c.11266C > T variant resides between the BIR and UBC domains. (**B**) Multiple sequence alignment across vertebrate species, demonstrating evolutionary conservation of the arginine residue at position 3756. High conservation underscores its structural and functional importance in BIRC6-mediated apoptosis regulation

The *BIRC6* gene, spanning 261,993 bp across 74 exons, encodes a 4,857-amino acid protein harboring two functional domains: the baculovirus IAP repeat (BIR) domain (residues 284–358) and the ubiquitin-conjugating (UBC) catalytic domain (residues 4,573–4,740) [66]. The homozygous variant (NM_016252.4: exon 55: c.11266C > T; p.Arg3756Cys; rs367790330) is located in the structurally critical linker region connecting the BIR and UBC domains, replacing a positively charged, hydrophilic arginine with a neutral, hydrophobic cysteine (Fig. 3A). Recent cryo-EM studies reveal that BIRC6 adopts a dimeric U-shaped conformation, in which the intervening segment orients BIR and UBC modules to facilitate the coordination of caspase binding with ubiquitin conjugation [43, 67, 68]. This substitution eliminates the wild-type positive charge and reduces side-chain volume, likely disrupting electrostatic interactions and BIR–UBC alignment required for coordinated caspase inhibition and ubiquitination [69]. Evolutionary conservation analysis showed strong preservation of Arg3756 across vertebrates (GERP + + score: 5.5; Fig. 3B), underscoring its functional importance within this connector segment.​ Regarding BIRC6’s established role in suppressing apoptosis through coordinated BIR-mediated caspase inhibition and UBC-mediated ubiquitination [43], we propose that p.Arg3756Cys perturbs this essential inter-domain communication, potentially impairing enzymatic activity, substrate recognition, or caspase regulation and thereby contributing to the observed premature ovarian insufficiency phenotype.

### Generation of *birc6*^*del12/del12*^ Zebrafish Model: CRISPR/Cas9 Targeting and Mutant Line Establishment

**Fig. 4:**
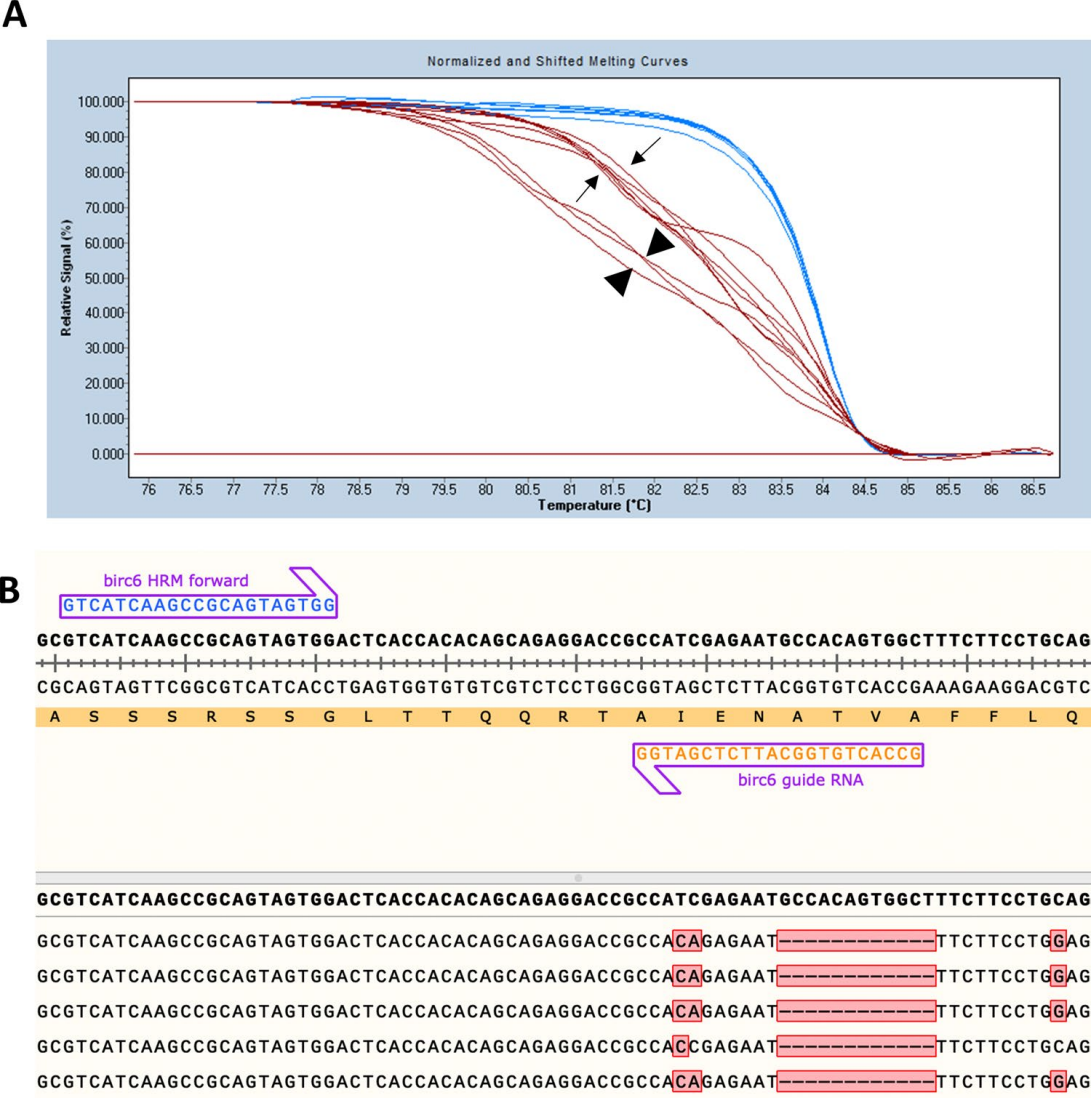
High-resolution melting analysis (HRMA) and Sanger Sequencing of *birc6*^*del12/del12*^ F2 generation fish tails. (**A**) HRMA graph showing distinct melting profiles for wild-type genomic DNA (blue, top), heterozygous mutant genomic DNA (red, middle, arrows), and homozygous mutant genomic DNA (red, bottom, arrowheads), indicating a shift in melting temperature associated with the *birc6*^*del12*^ mutation. (**B**) Sanger sequencing results of *birc6*^*del12/del12*^ homozygous mutant fish, showing the 12-nucleotide deletion aligned with the wild-type AB strain reference genome (GRCz11 assembly, NC_007128.7). Sequence alignment was performed using SnapGene software

Given the identification of a homozygous *BIRC6* variant (NM_016252.4: exon55: c.11266C > T: p.Arg3756Cys; rs367790330) in all affected family members, we generated a *birc6* knockout zebrafish model to investigate its functional relevance to POI. The zebrafish birc6 ortholog (ENSDARP00000128806) shares 78% amino acid identity with human BIRC6 (ENSP00000393596), based on Ensembl pairwise protein alignment (release 115) with conserved functional domains. A single-guide RNA (sgRNA) targeting exon 55 of *birc6* was co-injected with Cas9 mRNA into one-cell-stage wild-type AB embryos. HRMA of genomic DNA from injected F0 embryos revealed aberrant melting profiles in approximately 85% of the screened individuals, indicating a high frequency of CRISPR-induced mosaic mutations at the target locus. These abnormal HRMA profiles, characterized by broadened and shifted melting curves relative to wild-type controls, are consistent with the presence of mixed mutant and wild-type alleles within individual embryos. F0 mosaic founders were subsequently outcrossed to wild-type AB adults, followed by HRMA and Sanger sequencing to identify heterozygous F1 carriers of a defined 12-bp deletion (Fig. 4A and B). F1 *birc6*^*del12/*+^ heterozygotes were intercrossed to generate F2 progeny, yielding Mendelian ratios of 25% *birc6*^*del12/del12*^ homozygous mutants, 50% heterozygotes, and 25% wild-type individuals. For phenotypic analysis, we retained 50% of homozygous mutants (female *birc6*^*del12/del12*^), which represent 12.5% of the total F2 offspring, as POI primarily manifests in females.

### Reproductive Dysfunction in *birc6*^*del12/del12*^ Mutants

**Fig. 5:**
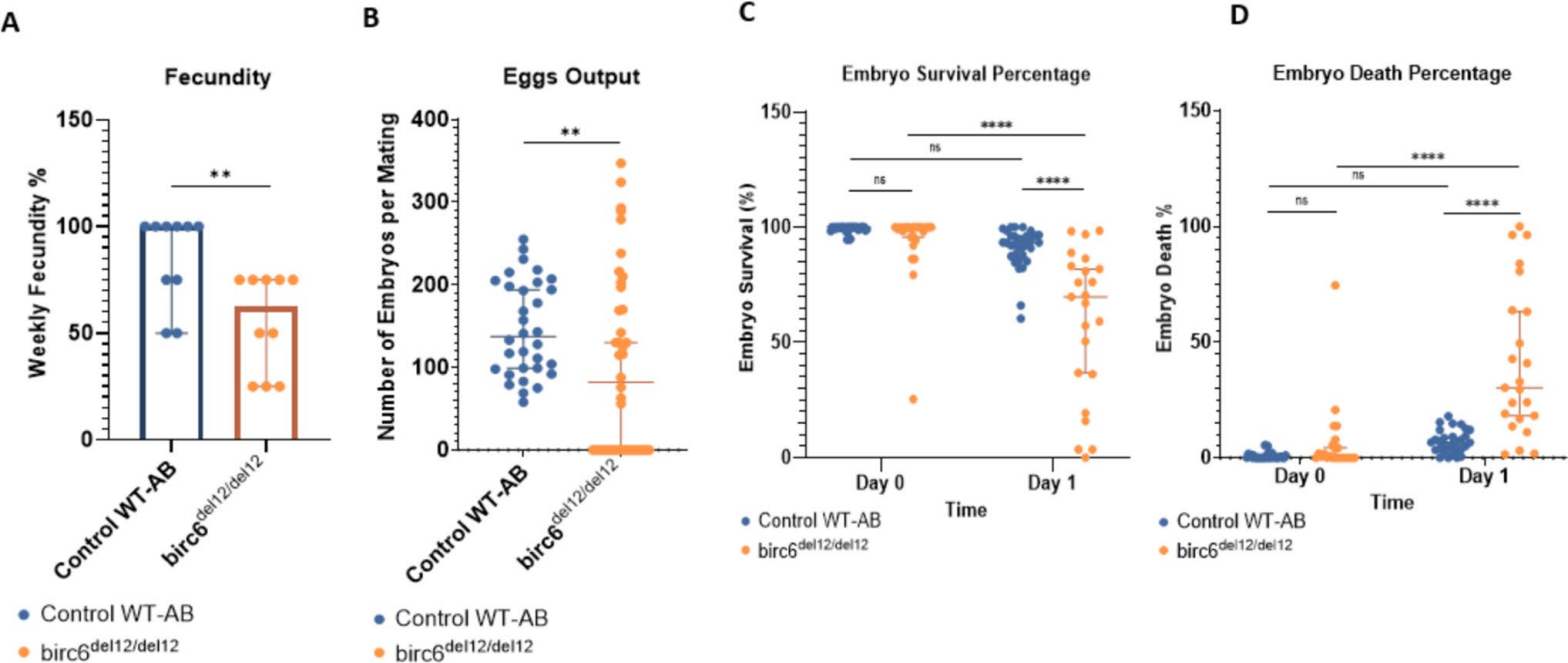
Reproductive performance of *birc6*^*del12/del12*^ zebrafish compared to wild-type controls. (**A**) Fecundity percentage (successful embryo-producing matings per total trials). (**B**) Embryos per clutch produced during a 1-h mating assay. *birc6*^*del12/del12*^ females (*n* = 6) exhibit significantly reduced fecundity compared to wild-type (WT; AB strain) females (*n* = 10). (**C**) Survival rates of embryos at 0- and 24-h post-fertilization (hpf). (**D**) Embryonic necrosis rates at 0 and 24 hpf, demonstrating elevated mortality in progeny derived from *birc6*^*del12/del12*^ females. Error bars represent mean ± SD. **p < 0.01, ****p < 0.0001; *ns* not significant

To evaluate the impact of the *birc6* loss-of-function on reproductive capacity, we conducted a 10-week mating assay comparing *birc6*^*del12/del12*^ homozygous mutant females (*n* = 6) with wild-type AB females (*n* = 10). Cohorts were paired with WT males, and three parameters were quantified weekly: (1) fecundity (percentage of mating events producing ≥ 1 embryo), (2) clutch size (total embryos per mating pair), and (3) 48-h embryo survival. Mutant females exhibited a 36% reduction in fecundity compared to WT-AB controls (Fig. 5A, *p* < 0.01). Among successful matings, *birc6*^*del12/del12*^ females produced 22% fewer embryos per clutch (WT: 147 ± 50.44 *vs.* mutant: 102.65 ± 93.63; Fig. 5B), with pronounced variability in clutch size (coefficient of variation: WT = 34.31% *vs.* mutant = 91.21%). Embryos from *birc6*^*del12/del12*^ females showed a 5.93-fold increase in necrosis at 24 h post-fertilization (WT: 6.92%3.86 *vs.* mutant: 41.03% 26.74; Figs. 5C and D). Notably, half of the *birc6*^*del12*^ mating attempts failed to produce embryos, and successful clutches exhibited irregular embryo counts (Fig. 5B). Besides, crosses between homozygous *birc6*^*del12/del12*^ males and females (*n* = 10 pairs) yielded no viable embryos across all trials (data not shown), demonstrating complete infertility in homozygous mutant pairs. These results establish that the *birc6* loss-of-function disrupts reproductive success through reduced fecundity, impaired embryo viability, and complete infertility in homozygous pairs, mirroring the reproductive deficits observed in the human POI phenotype.

### Ovarian Morphology and Egg Quality in *birc6*^*del12/del12*^ Mutants

**Fig. 6:**
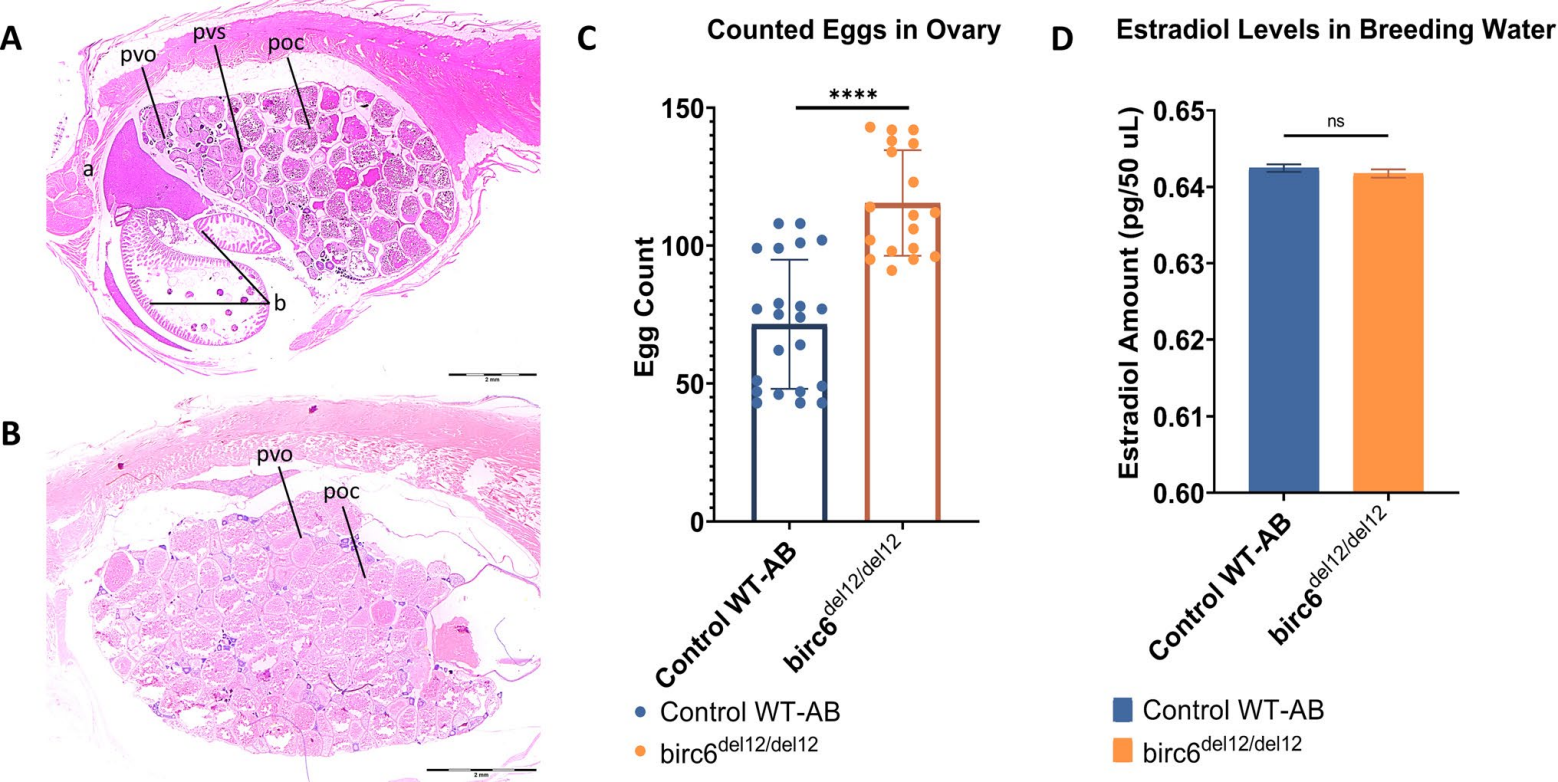
Ovarian histology and systemic estradiol levels in *birc6*^*del12/del12*^ zebrafish. (**A**) Hematoxylin and eosin (H&E)-stained ovarian section from wild-type (WT; AB strain) zebrafish, illustrating intact follicular architecture: liver (a), intestine (b), previtellogenic oocytes (pvo), primary oocytes (poc), and perivitelline space (pvs). (**B**) H&E-stained ovarian section from *birc6*^*del12/del12*^ mutants, showing retained follicular compartments but reduced pvs width (arrowheads), indicative of cytoplasmic maturation defects. (**C**) Quantification of total oocytes per ovarian section. *birc6*^*del12/del12*^ mutants exhibit a 1.6-fold increase in oocyte count compared to WT (****p < 0.0001), consistent with an increase in egg production accompanied by reduced gamete quality. Each dot represents an independent biological replicate (WT: *n* = 3; mutant: *n* = 6). (**D**) Systemic estradiol levels measured in breeding water post-mating assay. No significant difference (ns) was observed between genotypes (WT: 0.642454 ± 0.00045 pg/mL vs. mutant: 0.641734 ± 0.00051 pg/mL; Student’s t-test), excluding hormonal imbalance as a contributor to infertility. Error bars: mean ± SD. Scale bar: 2 mm

To investigate the in vivo effects of *birc6* loss-of-function, we quantified oocyte numbers and assessed morphological features in paraffin-sectioned ovaries from adult WT-AB and *birc6*^*del12/del12*^ females (*n* = 6 per group). Histological analysis revealed a 1.6-fold increase in total oocyte count in mutant ovaries compared to WT-AB controls (WT: 75.3 ± 21.8 *vs.* mutant: 114.2 ± 18.2; *p* < 0.001, Figs. 6A-C). Paradoxically, this elevated oocyte production coincided with structural abnormalities indicative of reduced oocyte quality. Notably, the *birc6*^*del12/del12*^ oocytes exhibited a minimized perivitelline space (Figs. 6A and B), a hallmark of defective cytoplasmic maturation linked to impaired embryo viability [70]. The inverse relationship between oocyte quantity and quality suggests a compensatory mechanism wherein *birc6*^*del12/del12*^ mutants overproduce oocytes to offset high rates of developmental failure, consistent with the reduced clutch sizes and elevated embryo necrosis observed in mating assays (Fig. 5A).

### *birc6*^*del12*^ Alters Fertility-Related Gene Expression Without Affecting Estradiol Levels

**Fig. 7:**
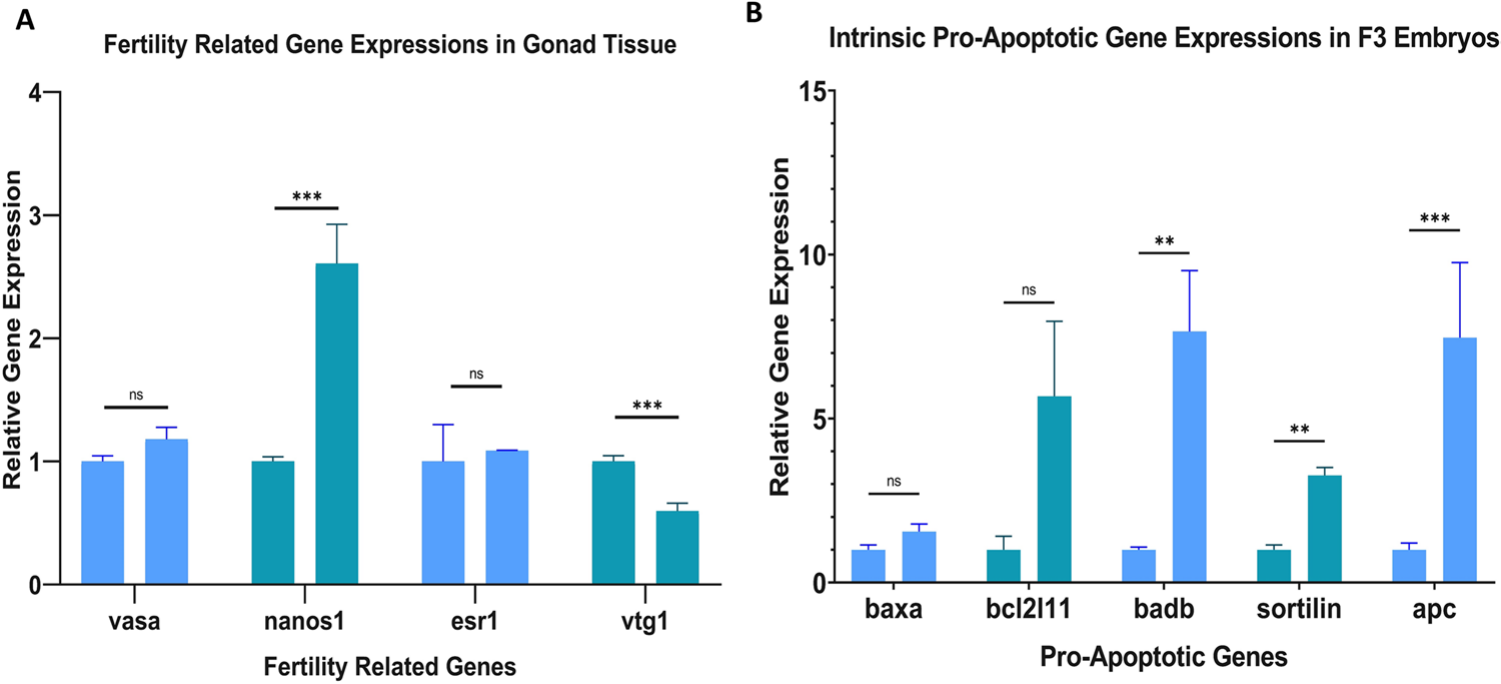
Transcriptional dysregulation of apoptosis- and fertility-associated genes in *birc6*^*del12/del12*^ zebrafish. (**A**) Quantitative RT-PCR (qRT-PCR) analysis of fertility-related genes in gonads of adult *birc6*^*del12/del12*^ mutants exhibits marked upregulation of *nanos1* (***p < 0.001), downregulation of *vtg1* (***p < 0.001), and unchanged *esr1* (ns). (**B**) qRT-PCR analysis of pro-apoptotic gene expression in F3 heterozygous *birc6*^*del12/*+^ embryos at 24 h post-fertilization (hpf). These embryos show significant upregulation of *badb* (***p < 0.001), *sortilin* (***p < 0.001), and *apc* (**p < 0.01) compared to wild-type (WT; AB strain) controls, indicative of apoptosis dysregulation. WT controls (left bars) and *birc6*^*del12/*+^ mutants (right bars) are normalized to *actb1* expression. Error bars: mean ± SD. Statistical significance assessed via Student’s t-test: **p < 0.01, ***p < 0.001, *ns* not significant

Egg production is closely related to hormonal balance [71]. To assess potential endocrine disruption, we quantified serum estradiol levels in adult WT-AB and *birc6*^*del12/del12*^ females (*n* = 4 per group) via ELISA. However, no significant difference in estradiol levels was observed between genotypes (WT: 0.64 ± 0.0005 pg/mL vs. mutant: 0.64 ± 0.0005 pg/mL; *p* = 0.0985, Fig. 6D), indicating that the reproductive deficits in *birc6*^*del12/del12*^ mutants are not attributable to systemic hormonal imbalance. Further investigation revealed altered expression of key fertility-associated genes in the gonads of *birc6*^*del12/del12*^ zebrafish (Fig. 7A). *Vasa* (*ddx4*), a protein interacting with intrinsic apoptosis pathway components and associated with ovarian development and maturation [72, 73], remains intact. In contrast, *nanos1*, an anti-apoptotic gene critical for germ cell survival [74–77], was upregulated 2.5-fold (*p* < 0.001), likely compensating for impaired *birc6* function. Downregulation of *vitellogenin 1* (*vtg1*; *p* < 0.001), essential for yolk synthesis and oocyte maturation [78–80], correlated with histological evidence of reduced yolk deposition (Fig. 6). Surprisingly, *estrogen receptor 1* (*esr1*) expression remained unchanged (*p* = 0.1487), aligning with unaltered estradiol levels and further excluding hormonal etiology [49, 81]. These results demonstrate that *birc6*^*del12*^-associated infertility arises from transcriptional dysregulation impacting oocyte quality and maturation, independent of systemic estrogen signaling. The compensatory upregulation of *nanos1* and downregulation of *vtg1* highlight a disrupted molecular axis governing germ cell maintenance and nutrient provisioning, which is consistent with the observed ovarian hyperproduction of low-quality oocytes.

### Pro-Apoptotic Gene Expression in F3 *birc6*^*del12/*+^ Heterozygous Embryos

The *birc6* acts as an anti-apoptotic protein. To assess the functional consequence of the *birc6* haploinsufficiency, we quantified pro-apoptotic gene expression via qRT-PCR in F3 *birc6*^*del12/*+^ embryos derived from heterozygous crosses (*n* = 6, 30 larvae/cross). Among five key apoptotic regulators analyzed, three—*badb*, *sortilin*, and *apc*—exhibited significant upregulation compared to wild-type controls (Fig. 7B). Among the tested genes, *baxa* serves as a key activator of the intrinsic apoptosis pathway, facilitating mitochondrial membrane permeabilization to initiate apoptosis [82]. *Bcl2l11, also known as bim,* is a pro-apoptotic member of the Bcl-2 family that antagonizes anti-apoptotic proteins to induce cell death. Similarly, another Bcl-2 family member, *badb,* promotes apoptosis by replacing baxa and binding to anti-apoptotic Bcl-2 proteins [83, 84]. *Sortilin*, a receptor protein, is involved in the cell trafficking of apoptotic signals [85]. Additionally, *apc* (adenomatous polyposis coli) is a crucial multifunctional protein, particularly in embryonic development, that regulates β-catenin degradation and induces cell death [86, 87]. The marked upregulation of *badb*, *sortilin*, and *apc* underscores the role of *birc6* in suppressing apoptosis, as heterozygous loss of a single allele disrupts this regulatory balance. The 5.2-fold induction of *apc*, a gene implicated in developmental apoptosis, aligns with embryonic necrosis observed in prior assays (Figs. 5C and D). However, as these results are based on transcript-level measurements of a limited gene set, they indicate dysregulation of apoptosis-associated gene expression rather than directly demonstrating functional activation of apoptotic pathways. Nevertheless, these results support a role for *birc6* in maintaining cell survival programs during early development and warrant further functional validation.

## Discussion

BIRC6 protein, a member of the inhibitor of apoptosis protein (IAP) family, is a critical regulator of cellular survival pathways. This ubiquitously expressed protein inhibits apoptosis through caspase suppression, ubiquitin-mediated degradation of pro-apoptotic factors, and modulation of autophagy. Structurally, BIRC6 harbors a ubiquitin-conjugating (UBC) domain essential for its enzymatic activity, as well as a baculovirus IAP repeat (BIR) domain that facilitates protein interactions [88, 89]. Functionally, it targets activated caspases (CASP-3, −6, −7, −8, and −9), pro-apoptotic serine protease HtrA2, and DIABLO/Smac, marking them for proteasomal degradation [39, 40]. Unlike other IAPs, BIRC6 uniquely inhibits both precursor and mature forms of caspase-9 and Smac, thereby effectively suppressing apoptosis initiation [90]. Furthermore, BIRC6 stabilizes cellular homeostasis by promoting p53 ubiquitination and degradation, indirectly attenuating mitochondrial apoptosis pathways. The indispensability of the BIRC6 in reproductive biology is underscored by murine models, where *Birc6* deficiency induces placental and yolk sac apoptosis, culminating in embryonic lethality [91, 92]. Recent studies highlight its dual role in balancing apoptosis and autophagy; BIRC6 not only inhibits caspase-9 but also facilitates LC3 ubiquitination to enhance autophagic flux [43]. These mechanisms are particularly salient in ovarian biology, where follicular atresia—a tightly regulated apoptotic process—determines oocyte pool longevity.

In this study, we identified a novel homozygous *BIRC6* missense variant (NM_016252.4: exon 55: c.11266C > T; p.Arg3756Cys; rs367790330) in a consanguineous Iranian family with autosomal recessive POI. The variant lies in a structurally critical linker region between the BIR and UBC domains, which is essential for coordinating caspase inhibition with ubiquitin conjugation through proper inter-domain alignment. The variant's impact on the charge of the wild-type residue and the reduction of side chain volume, coupled with the evolutionary conservation of arginine at position 3756 (GERP + + score: 5.5; Fig. 3B), suggests functional disruption of ubiquitin ligase activity or interactions with collaborator proteins through impaired BIR-UBC alignment. Computational pathogenicity predictions were discordant, reflecting the variant’s complex biophysical impact: substituting a charged arginine with a hydrophobic cysteine likely perturbs electrostatic interactions critical for substrate recognition or catalytic activity [69]. POI in this family manifested as secondary amenorrhea, elevated gonadotropins (FSH: 36.4 IU/L; LH: 21.1 IU/L), and decreased AMH (0.3 ng/mL), consistent with follicular depletion (Table 2). The absence of the variant in ethnically matched controls (Iranome database) and its low population frequency (gnomAD MAF: 0.00002752) further support its pathogenic potential. Segregation analysis confirmed an autosomal recessive inheritance pattern, with homozygosity detected in the affected sisters (Fig. 2). Notably, an unaffected homozygous male sibling suggests possible sex-limited expression (given POI's ovarian etiology), incomplete penetrance, or contribution of additional modifiers. This pattern reflects variable expressivity typical of recessive POI.

**Table 2:** Clinical findings, genetic tests and hormonal assessments

Subjects (see Fig. 1)	III-4	III-5	III-6	III-9
Age (y)	49	46	44	38
Age of menopause (y)	36	30	38	36
Menstruation type	Amenorrhea	Amenorrhea	Amenorrhea	Amenorrhea
History of infertility	No	Yes	No	Yes
Type of infertility	-	Primary	-	Primary
External genitalia	Normal	Normal	Normal	Normal
FMR1 CGG repeats	32 and 35	29 and 36	24 and 35	32 and 38
Karyotype	46, XX	46, XX	46, XX	46, XX
FSH (IU/L)	51.1	87	45.3	36.4
LH (IU/L)	24.1	32.7	19.5	21.1
AMH (ng/mL)	0.1	0.05	0.04	0.3
Estradiol (pg/mL)	41.5	32.6	66.7	87.1
BMI	Normal	Normal	Normal	Normal
Intellectual ability	Normal	Normal	Normal	Normal
Ultrasonography	Decreased ovarian size No follicle	Decreased ovarian size No follicle	Decreased ovarian size No follicle	Decreased ovarian size No follicle

The involvement of BIRC6 in ovarian function has been indirectly suggested through investigations of associated IAPs. For instance, NAIP (BIRC1) and XIAP (BIRC4) safeguard granulosa cells and oocytes by neutralizing caspases, thereby preserving follicular integrity [93–95]. Granulosa cell dysfunction—a hallmark of POI—disrupts FSH-mediated estradiol production and oocyte maturation [96]. Similarly, during perinatal development, murine oocytes exhibit sustained expression of anti-apoptotic genes (*Birc5, Birc6*), while simultaneously downregulating pro-apoptotic factors (*Trp53, Casp9*), thereby ensuring follicular survival [97]. Our findings are consistent with these mechanisms and suggest that *BIRC6* deficiency accelerates follicular atresia through dysregulated apoptosis in granulosa cells or oocytes. Collectively, our data position BIRC6 within the IAP family–mediated network regulating ovarian apoptosis, alongside XIAP, which has been implicated in POI through miR-23a–induced granulosa cell death [98]. Proof-of-concept studies in chemotherapy-induced ovarian damage further demonstrate that pharmacological inhibition of apoptotic pathways—such as imatinib-mediated blockade of the c-Abl → p63/p73 → Bax axis or ICRF-187–mediated prevention of DNA damage—can rescue primordial follicles, thereby establishing apoptosis pathway modulation as a feasible fertility preservation strategy [99, 100]. In the context of genetic POI, stabilization or functional preservation of *BIRC6* may similarly contribute to maintaining the follicular reserve, potentially complementing established approaches such as oocyte cryopreservation, although etiology-specific validation will be required.

To validate *BIRC6* loss-of-function, we generated a *birc6* knockout zebrafish model targeting exon 55. Homozygous *birc6*^*del12/del12*^ female zebrafish exhibited profound reproductive deficits, including reduced fecundity (Fig. 5A), diminished clutch sizes (Fig. 5B), and elevated embryonic death (Figs. 5C and D). Paradoxically, mutant females showed an increase in egg production, accompanied by morphological abnormalities in oocytes (Fig. 6A–C), suggesting dysregulated ovarian homeostasis rather than enhanced reproductive fitness. While this may reflect a compensatory response to germ cell loss, alternative explanations should also be considered, including altered timing of follicular atresia, developmental delay in oocyte maturation, or transient compensatory folliculogenesis that ultimately fails to sustain oocyte quality. Such mechanisms could contribute to the apparent increase in egg output despite impaired oocyte quality and progressive follicular loss. Mechanistically, *birc6*^*del12/del12*^ mutants exhibited transcriptional dysregulation of fertility-associated genes: downregulation of *vtg1* (critical for yolk provisioning; *p* < 0.001) and upregulation of *nanos1* (anti-apoptotic; *p* < 0.001) (Fig. 7A). These changes occurred despite normal systemic estradiol levels (Fig. 6D), excluding endocrine disruption as a primary etiology. Instead, apoptosis dysregulation was evident in heterozygous embryos, where haploinsufficiency induced overexpression of pro-apoptotic genes (*badb*, *sortilin*, and *apc*; Fig. 7B). The 5.2-fold induction of *apc*—a regulator of developmental apoptosis—aligns with the embryonic necrosis observed in mating assays (Figs. 5C and D). However, as these conclusions are based on transcript-level data, they should be interpreted as indicative of perturbed apoptotic regulation rather than definitive evidence of increased apoptotic activity.

Collectively, our data suggest that loss of Birc6 function destabilizes ovarian homeostasis, likely through impaired regulation of cell survival pathways. The observed increase in egg output may reflect a transient or maladaptive response to germ cell stress, potentially arising from altered atresia dynamics or compensatory follicle recruitment, but ultimately culminating in poor oocyte quality and reproductive failure. Taken together, these findings identify *BIRC6* as a novel candidate gene for familial POI and establish the *birc6*^*del12*^ zebrafish line as a tractable model to explore how apoptotic dysregulation contributes to ovarian insufficiency and fertility loss.

### Limitations and Future Directions

While the zebrafish *birc6*^*del12*^ model provides valuable in vivo evidence linking BIRC6 dysfunction to ovarian failure, several limitations should be acknowledged. Most importantly, this model represents a loss-of-function allele, whereas the human condition is associated with a missense variant (BIRC6 p.Arg3756Cys). Knockout models are informative for defining gene function, but they may not fully recapitulate the molecular consequences of missense mutations, which can retain partial activity or selectively disrupt specific protein–protein interactions rather than causing complete functional ablation [101, 102]. Given the multifunctional nature of BIRC6 in ubiquitin-mediated proteostasis and apoptosis regulation, complete loss of *birc6* may engage compensatory pathways or developmental stress responses that differ from those elicited by a single amino acid substitution.

In addition, although zebrafish share conserved regulators of folliculogenesis and apoptotic signaling with mammals, important species-specific differences exist in ovarian organization, follicle reserve establishment, endocrine feedback, and reproductive lifespan [71, 73, 103]. Therefore, phenotypes such as altered egg production or embryonic viability should be interpreted as indicators of disrupted ovarian homeostasis rather than direct analogs of human follicle depletion dynamics in POI. Moreover, the mechanistic interpretation remains limited, as the present study does not yet resolve which ovarian cell types are primarily affected by Birc6 loss or how cell-type-specific apoptotic responses contribute to the observed phenotypes. Addressing these questions will require future studies incorporating ovarian cell-type markers, lineage analyses, and quantitative histological approaches.

Another limitation is the relatively modest sample size used in fertility assays, which reflects the limited availability of homozygous mutant females. Although the phenotypes observed were robust and supported by power analysis, larger cohorts will be important in future studies to better capture biological variability and strengthen statistical confidence. In addition, while increased embryo necrosis was consistently observed, we cannot fully distinguish whether this reflects fertilization failure or post-fertilization developmental defects, and future experiments incorporating fertilization markers or early cleavage-stage assessments will be required to resolve this distinction.

Despite these limitations, the model consistently implicates apoptosis-driven follicular atresia, rather than endocrine insufficiency, as a central mechanism downstream of BIRC6 loss, in line with the established anti-apoptotic role of BIRC6/BRUCE in regulating caspase activation and mitochondrial integrity [39, 92, 104]. From a translational perspective, these findings suggest that targeting intrinsic apoptotic pathways may represent a complementary therapeutic strategy for fertility preservation in genetically defined subsets of POI, beyond hormone-based interventions [105, 106]. Future studies employing variant-specific knock-in models and mammalian systems will be essential to refine genotype–phenotype relationships and assess therapeutic feasibility.

## Supplementary Information

Below is the link to the electronic supplementary material.

Supplementary file1 (DOCX 248 KB)

## Data Availability

The data supporting the findings of this study are available from the corresponding author upon reasonable request.
