# Supplementary material for: A Novel BIRC6 Variant Impairs Apoptotic Regulation in Familial Premature Ovarian Insufficiency: Functional Validation in a CRISPR/Cas9 Zebrafish Model
Source: Reprod Sci. 2026 May 4;33(6):1170–88. doi: 10.1007/s43032-026-02088-6 (PMC13328166; doi:10.1007/s43032-026-02088-6)
Supplement: Supplementary file 1 — Supplementary file1 (DOCX 248 KB) [file 43032_2026_2088_MOESM1_ESM.docx]

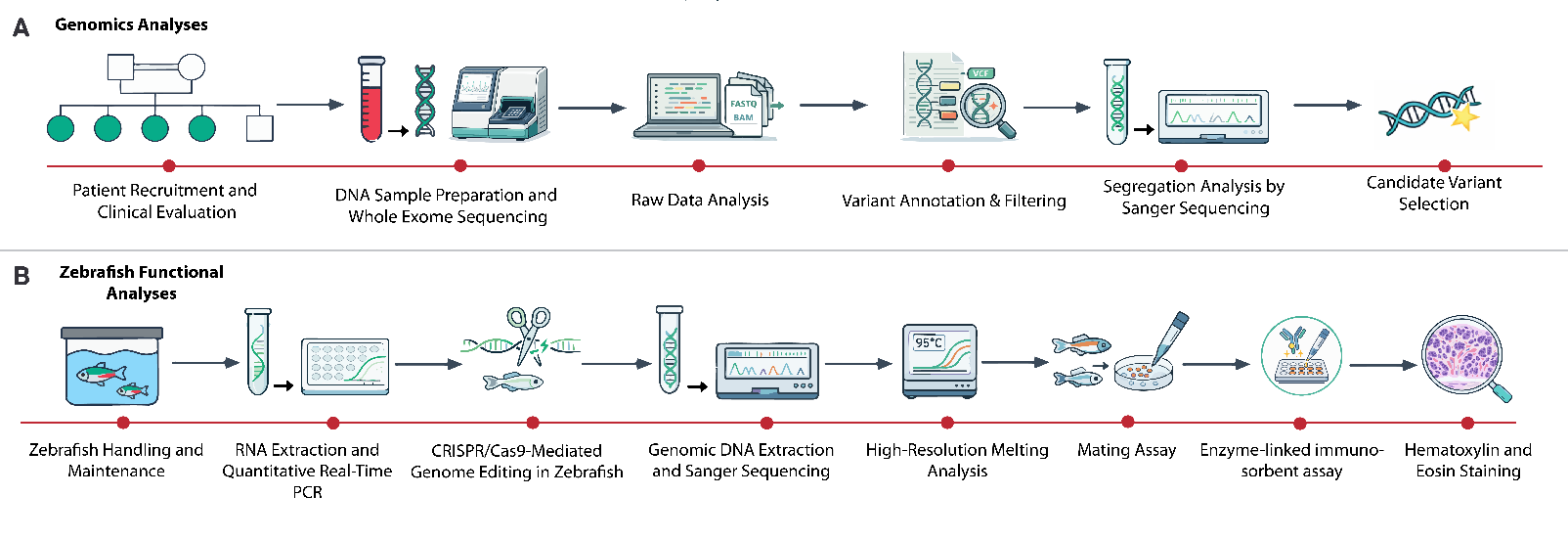


**Supplementary Figure S1.** **Schematic workflow of genetic analyses and zebrafish experiments.** The workflow was created in Adobe Illustrator software, with icons representing individual steps generated using AI tools. Labels, arrows, and the overall arrangement of the workflow were added manually to clearly illustrate the experimental timeline. All AI-generated elements are used solely for illustration purposes.
